# Experimental study on the vertical load-bearing performance of single piles and pile groups in saline soil foundations

**DOI:** 10.1371/journal.pone.0352551

**Published:** 2026-08-25

**Authors:** Xiaolin Cao, Lianbin Xue, Fengxi Zhou, Guoliang Dai

**Affiliations:** 1 School of Civil and Hydraulic Engineering, Lanzhou University of Technology, Lanzhou, Gansu, China; 2 School of Civil Engineering, Southeast University, Nanjing, Jiangsu, China; China Construction Fourth Engineering Division Corp. Ltd, CHINA

## Abstract

To investigate the vertical bearing performance of single and group piles in layered saline soil, a series of indoor model tests were conducted. A multi-pile theoretical calculation model correlating soil salinity, pile–soil shear stiffness, and vertical bearing capacity was established, and corresponding calculation methods for single and group piles were further proposed. The *Q*-*S* curves, pile top stiffness, and pile group interaction coefficients under different salinity conditions were acquired via model testing, and the influencing mechanism of saline soil salinity on pile group interaction was systematically analyzed. Test-derived stiffness and pile group efficiency coefficients were compared with theoretical calculations to validate the reliability of the experimental results. Consistent with the unified logical framework of the manuscript’s Results and Discussion sections, this study clarifies the differential effects of soil salinity on pile bearing behavior: varying salt concentrations do not change the overall evolutionary trend of pile *Q*-*S* curves, whereas they impose a significant quantitative influence on ultimate bearing capacity. Under identical loading conditions, increasing soil salinity effectively reduces the settlement of both single and group piles and enhances pile top stiffness, thereby shifting the *Q*-*S* curves toward lower settlement values. Obvious pile–pile interaction exists in pile group foundations, while the stiffness of group piles does not exhibit a simple multiple relationship with that of single piles. Additionally, the pile group effect gradually weakens with the increase in the number of foundation piles.

## 1. Introduction

Saline soils are extensively distributed across China, covering approximately 36.9 million hectares and accounting for 5% of the nation’s available land resources. These soils primarily concentrate in four zones: Northwest, North, and Northeast China, as well as the eastern coastal regions. Western provincial-level regions including Shaanxi, Gansu, Ningxia, Qinghai, Inner Mongolia and Xinjiang occupy over 69% of China’s total saline soil area, featuring high salinity and strong corrosivity [1]. Subjected to freeze-thaw coupling, such soils suffer severe deterioration of pile-soil interfacial mechanical performance, which degrades the bearing capacity and long-term durability of pile foundations. Driven by the advancement of the Belt and Road Initiative, a growing number of infrastructure constructions have been launched along relevant routes. Accordingly, the safe service and long-term stability of pile foundations in saline soil regions have emerged as critical research topics requiring urgent solutions in geotechnical engineering.

Based on the current research status at home and abroad, the vertical bearing capacity and settlement characteristics of pile foundations remain the core issues in pile foundation engineering design. To accurately predict pile foundation settlement, many scholars have proposed various methods for calculating pile foundation settlement, including the load transfer method [2–11] the shear displacement method [12–15] the elastic theory method [16,17] and the numerical analysis method. It was [2] who first proposed the theoretical analysis framework of the load transfer method via experimental investigations and established the classical *t*-*z* curve model. Within the load transfer theory, [3–7] have successively improved the method. The load transfer method is accurate but cannot be extended to group piles because it does not consider the continuity of the soil. Subsequently [8,9] combined the Boussinesq solution to determine the equivalent stiffness of the soil around the pile and at the pile tip, and constructed a settlement calculation model for group piles. [10] introduced the hyperbolic tangent function and proposed an analysis method for the pile-soil interaction of axially loaded energy piles based on the load transfer method. [11] proposed an analytical algorithm for the axial load-settlement curve of piles based on the load transfer method.

At present, most of the research on pile foundations based on the load transfer method simplifies the interaction between the soil at the top of the pile and the pile itself as a spring model. The model parameters are mainly determined by inversely inferring from the measured pile top reaction-displacement curve. This method has obvious drawbacks. The spring parameters cannot reflect the actual physical properties of the soil at the pile top, so each pile foundation needs to undergo on-site tests. The method has poor universality and generalizability, and its application effect is particularly limited in the parameter calculation and performance analysis of group pile foundations. To make up for the above deficiencies, the virtual soil pile model has gradually formed and been continuously optimized and improved. Among them, reference [18] combined this model with the ideal elastic-plastic transfer function and used the load transfer method to derive the pile foundation load-settlement relationship considering the plastic deformation of the soil around the pile; reference [19] based on the dynamic equilibrium principle, established the settlement calculation method for the virtual soil piles of group piles. Sodic soil has strong corrosive properties. The chemical reaction between it and the pile foundation concrete will significantly reduce the bearing capacity of the pile foundation. Specifically, it leads to the attenuation of the lateral resistance and the end resistance of the pile, a decrease in the structural strength of the pile body, and accelerates the deterioration of the durability of the pile foundation, which has a significant impact on the safe and stable operation of the pile foundation project.- Therefore, conducting research on the interaction mechanism between sodic soil and pile foundations has important engineering value. Currently, many scholars have conducted studies on the corrosion and mechanical properties of pile foundations in sodic soil areas: Literature [20] clarified the damage mechanism of sulfate crystalline erosion on the underground concrete pile foundation structure; Literature [21] constructed a comprehensive numerical simulation method for the damage and deterioration of concrete piles under sulfate erosion environment; Literature [22] systematically summarized the corrosion laws of sodic soil in Qinghai Gela Ba salt lake area on concrete structures. Regarding frozen and thawed sodic soil and frozen soil sites for pile foundations, Literature [23] analyzed the influence of soil parameter changes on the reliability of vertical bearing capacity of bridge pile foundations under different thicknesses of frozen soil layers, and Literature [24] through indoor model tests, explored the differences in vertical bearing characteristics between permanent frozen soil and non-frozen soil site bridge piers.

In conventional non-saline soil sites, the research system related to pile foundations has become mature. Reference [25] conducted model tests of single piles in saturated sandy soil and groups of piles with different spacing, and clarified the influence laws of pile spacing on the side friction resistance of piles, the end resistance of piles, the reaction force of the subsoil at the base of the platform, and the group pile effect; Reference [26] combined with the vertical dynamic tests of pile foundations on site, established vertical calculation models for single piles and groups of piles in multi-layer soil bodies in non-saline sites, improved the theoretical system of pile foundation dynamic response, and provided theoretical support for the analysis of the dynamic characteristics of pile foundations in conventional sites.

Compared with the mature research on non-saline soil pile foundations, the research on the mechanical deformation characteristics of saline soil site pile foundations started relatively late. Due to the special physical and chemical properties of saline soil, the force mechanism, deformation laws, working performance of pile foundations in saline soil sites differ significantly from those of conventional soil pile foundations. Therefore, scholars at home and abroad have carried out a large number of targeted special studies. Reference [27] abandoned the conventional research ideas for non-saline soil pile foundations and, relying on the COMSOL numerical simulation platform, explored the influence laws of pile diameter, pile spacing, pile arrangement mode and pile number on the heat transfer performance of pile groups in saline soil. Reference [28] took the saline lake sulfate-chloride composite saline soil site as the research background and, through a series of tests of long and short pile groups composite foundations, analyzed the influence characteristics of pile spacing and soil salt content on the coordinated deformation of the foundation, the pile-soil stress ratio and the group pile effect. This further supplemented the research results of pile foundations in special saline soil sites.

In summary, current research on the vertical bearing performance of pile foundations in salt-affected soil areas mainly focuses on corrosion and frost heave effects. Systematic exploration of pile-soil mechanical interaction, pile foundation interaction factors, and group pile effects is still insufficient. This paper simulates the real salt-affected soil environment through indoor scaled model tests and analyzes the direction of “salinity-shear stiffness-vertical bearing capacity of multiple piles.” Based on the virtual soil pile method, a simplified calculation method for vertical static load action in salt-affected soils is established for single piles, two piles, four piles, six piles, and nine piles. The effects of different salinities on the vertical bearing performance of various pile foundations are systematically explored by comparing experimental and theoretical results.

## 2 Model test design

### 2.1 Model pile geometry and material parameters

**Fig 1:**
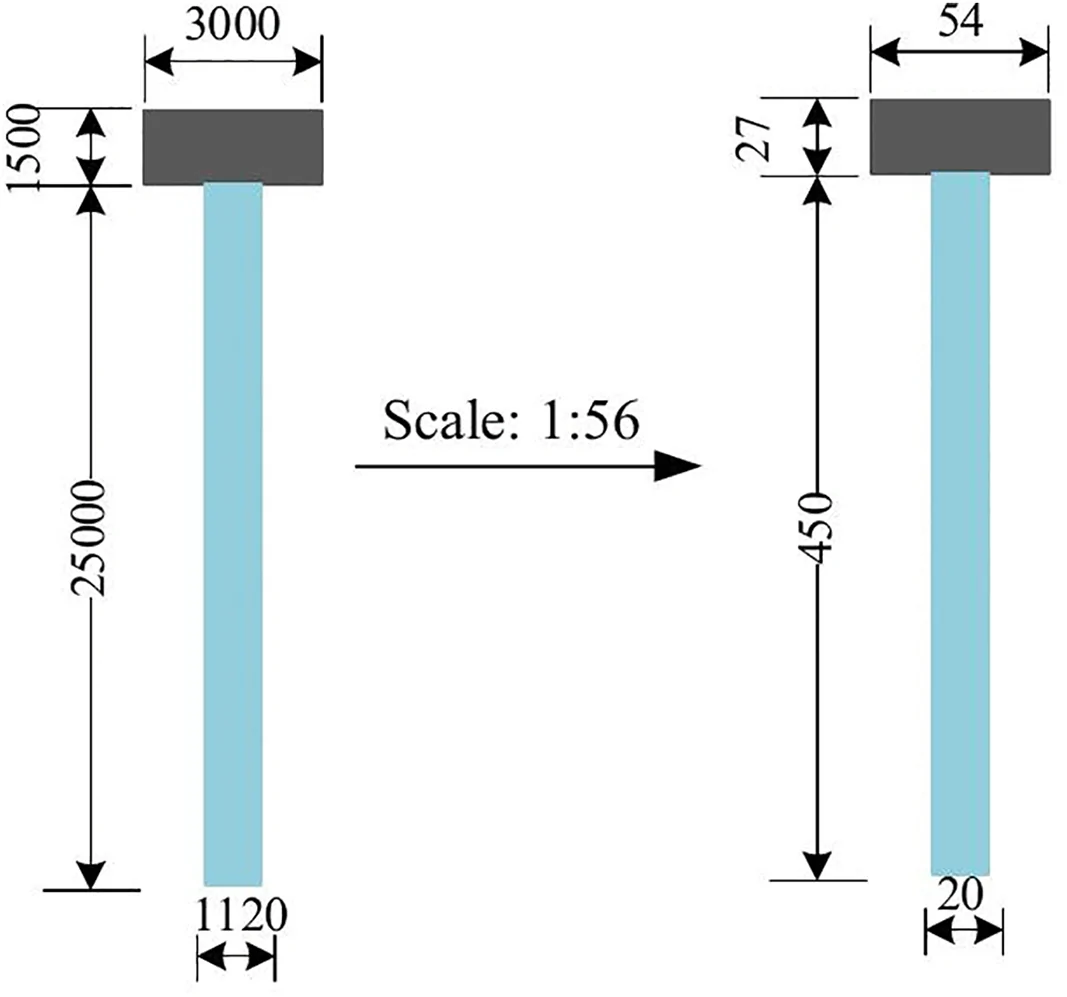
Prototype and dimensions of pile foundation (unit: mm).

The indoor test model is based on the pile foundation of a bridge in a region with long-term salt-affected soil. Its structural form and dimensions are shown in Fig 1 (this paper takes the double-pile foundation as the prototype and extends it to four-pile, six-pile, and nine-pile foundations on the basis of the double-pile foundation). The pile length is 25 m, and the pile diameter is 1.12 m. The model pile foundation part is a single pile, double pile, four-pile, six-pile, and nine-pile foundation made at a scale of 1:56 of the prototype, as shown in Fig 1. The pile length and diameter are 0.45 m and 0.02 m, respectively. The cap part is simplified to serve only as a loading platform for the test pile foundation.

**Fig 2:**
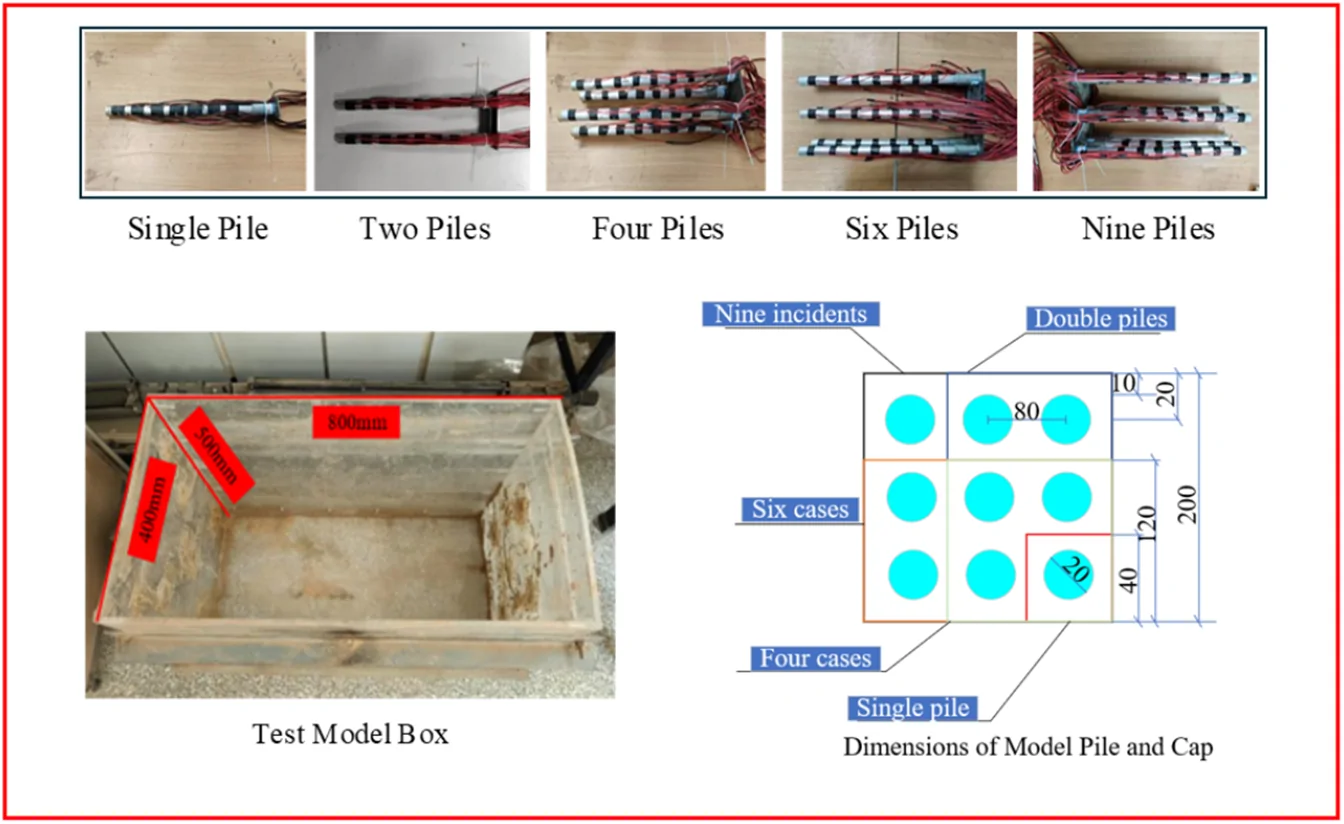
The size and layout of the model test set (unit: mm).

Model tests were carried out in the Structural Laboratory Hall, School of Civil and Water Resources Engineering, Lanzhou University of Technology. Multi-stage vertical static load tests were performed on 1 × 1, 1 × 2, 2 × 2, 2 × 3 and 3 × 3 pile group foundations in saline soils with salinity contents of 0%, 3% and 6%. According to the pile driving influence range in Ref. [29], the model box boundary shall be at least four times the pile diameter to avoid boundary effects. The adopted 10 mm-thick transparent acrylic model box has dimensions of 400 mm × 800 mm × 500 mm, which satisfies the size requirement for eliminating boundary effects (Fig 1). Hollow aluminum tubes were used as model piles, with a length of 450 mm, an outer diameter of 20 mm, an inner diameter of 18 mm and a wall thickness of 1 mm. The pile morphology is displayed in Fig 2, and relevant material parameters are listed in Table 1.

**Table 1:** Material parameters of aluminum pipe.

Material name	Elastic modulus	Poisson’s ratio
Aluminum Tube	71GPa	0.25
Yield Strength	Tensile Strength	Elongation at Break
455MPa	524MPa	11%

The cap is a rigid plate with a thickness of 5 mm. The dimensions between each pile and the cap are shown in Fig 2. Based on the research findings of [30] the stiffness of the test cap can be regarded as a rigid body, and its deformation under the action of pile pressing load can be neglected. According to this, it is assumed that the vertical load received by the group piles during the loading process is evenly distributed, that is, the load at the top of each pile foundation is the same.

### 2.2 Strain gauge layout and data acquisition

**Fig 3:**
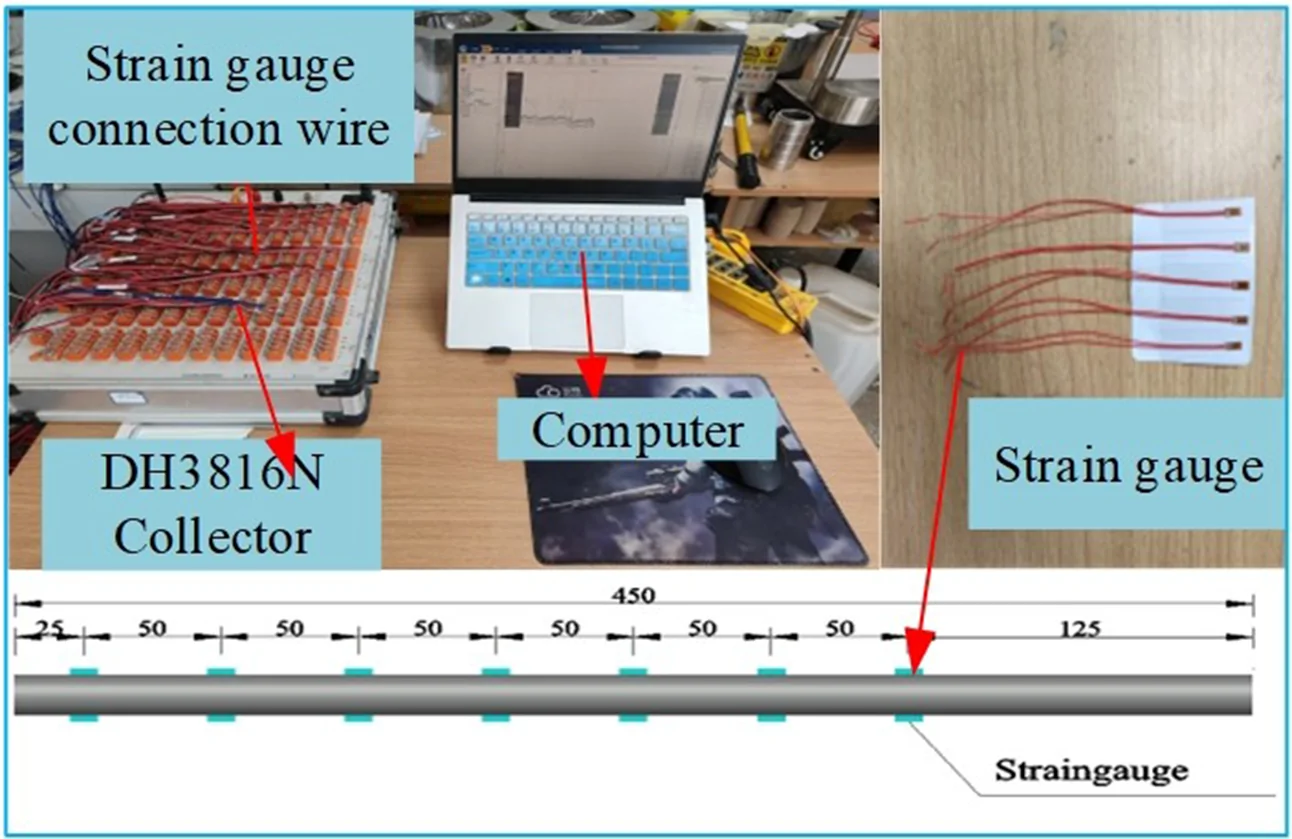
Model test equipment.

The resistance strain gauge used in this test is model BX120−3AA, with a grid length × grid width of 3 mm × 2 mm, a resistance range of 120.0 ± 0.2 Ω, a sensitivity coefficient of 2.06 ± 1%, and an accuracy class of A. During the entire loading process of the test, the data from the strain gauges were collected using the static strain testing system DH3816N. The strain gauges and the data acquisition instrument are shown in Fig 3.

Measured quantities in the model test covered pile top displacement, pile section strain, pile tip resistance and pile cap base soil pressure. Pile strain signals were captured by the DH3816N static strain tester (Fig 3) and recorded via supporting software. The LY-350 strain-type earth pressure cell (Φ17 mm) linked to the same strain tester was used to monitor pile tip resistance, while high-precision dial indicators recorded pile top settlement.

### 2.3 Saline soil

The test saline soil was prepared using natural loess collected from Qilihe District, Lanzhou. Following the procedure in Ref. [31], soil specimens were repeatedly rinsed with deionized water for desalination, until the electrical conductivity decreased to 0.001 s·m^-1^. After 24 h of static placement, clay particles remained suspended without supernatant clarification, and the desalination process was terminated. The treated soil was air-dried at ambient temperature for two weeks to a moisture content below 2%, gently ground via a porcelain mortar, and sieved through a 0.5 mm mesh to eliminate impurities. For test accuracy, the sieved loess was further air-dried for one more week to achieve a zero-moisture state.

Loess sieved through a 0.5 mm mesh was adopted in this test. The required mass of loess and anhydrous Na_2_SO_4_ crystals were determined according to the model box volume, to prepare saline soil specimens with salinity levels of 0%, 3% and 6%. The saline soil was filled in five equal layers of 100 mm thickness. The filling mass per layer was calculated based on the soil parameters in Table 2, and each layer was compacted repeatedly by an indoor lightweight compactor to the designed elevation. After layered filling and compaction, the soil was kept static for over 24 h for self-weight consolidation, thereby guaranteeing homogeneity of all soil specimens.

**Table 2:** The soil parameters of this experimental model are as follows.

Dry density	Natural density	Moisture content	Salinity /*w*	Salt type
1.55g/cm^3^	1.61g/cm^3^	7.0%	0%、3%、6%	Anhydrous Na_2_SO_4_ Crystalline Salt

### 2.4 Test plan and loading method

Due to the high clay content in the soil used in this test, the soil was consolidated for no less than 24 hours before the test began. The test procedure can refer to the “Code for Design of Building Foundation” (GB50007−2011) [32]. First, the ultimate bearing capacity of the pile was determined through a preliminary test, and then the loading test was conducted. After each load level was applied, the next level was applied after the pile settlement stabilized. The loading termination conditions include: 1) The pile top settlement is five times that of the previous level. 2) The settlement has not stabilized after more than one hour. 3) The pile top settlement drops sharply and cannot be accurately read.

## 3 Determination of the Shear model for saline soil

At a constant moisture content, increasing salt concentration first elevates the pore fluid concentration of saline soil. Once the pore solution reaches saturation, excess salt precipitates as crystals that fill and cement soil pores, reinforcing the soil skeleton. This process increases the internal friction angle and cohesion, thereby improving the shear strength with rising salt content. Experimental results from Ref. [33] indicate that the shear modulus of Na_2_SO_4_ saline soil increases monotonically with salt content under a constant confining pressure. For soils with a fixed salt content, however, the shear modulus is predominantly governed by confining pressure and void ratio. Furthermore, Ref. [34] reported a reversal in the strength variation trend when the Na_2_SO_4_ content exceeds 2%. Notably, conventional models are difficult to extend to other single salts or mixed saline soils. Based on the shear modulus determination method proposed in Ref. [35], this study incorporates the influences of confining pressure and void ratio to establish a modified shear modulus calculation formula.


$$\begin{array}{c} G_{\text{0}}=AF\left(e\right)(\frac{\sigma^{\prime }}{P_{a}}{)}^{n} \end{array}$$ (1)

In the formula,*σ′* is the confining pressure; *P*_*a*_ is the standard atmospheric pressure, taken as 100 kPa; *F(e)* is a function of the void ratio. According to the research by Iwasaki and Tatsuoka, for fine-grained soil, $F\left(e\right)=\frac{(2.17-e{)}^{\text{2}}}{1+e}$. Considering the complexity of the changes in void ratio due to the hydration and crystallization of Na_2_SO_4_, [33] combined the method proposed by [35] for determining the shear modulus and provided a revised calculation method for the shear modulus of saline soil.

Where: *A* and *n* are fitting parameters, determined based on the experimental results by [36] as shown in the following equation:


$$\begin{array}{c} G_{\text{0}}=AF\left(Na_{\text{2}}SO_{\text{4}}\right)F\left(e\right)+B(\frac{\sigma^{\prime }}{P_{a}}{)}^{n} \end{array}$$ (2)

In the formula, *F(Na_2_SO_4_)* is the sulfate function, and its expression is as follows, based on the variation of *G0* with different salt contents:


$$\begin{array}{c} F\left(Na_{\text{2}}SO_{\text{4}}\right)=e^{\frac{w\left(Na_{\text{2}}SO_{\text{4}}\right)}{-\beta_{\text{1}}}} \end{array}$$ (3)

Where: *ѡ* (*Na_2_SO*_*4*_) is the content of Na_2_SO_4_. *β1* and *B* are fitting parameters, which can be determined based on the experimental results by Ding Shuyun et al, $\sigma^{\prime }=\sigma_{x}=\sigma_{y}=k\sigma_{z}$, *k* is the soil pressure coefficient, which can be obtained from the Poisson’s ratio v of the model saline soil.


$$\begin{array}{c} k=\frac{\nu }{1-\nu } \end{array}$$ (4)

The interconversion relationship between the shear stiffness coefficient λ and the shear modulus is as follows:


$$\begin{array}{c} \nu =\frac{\lambda }{2(G_{\text{0}}+\lambda )} \end{array}$$ (5)

The calculation formula for the shear stiffness coefficient is obtained as follows:


$$\begin{array}{c} \lambda =\frac{2G_{\text{0}}\nu }{1-2\nu } \end{array}$$ (6)

The relationship between the pile-side shear stiffness coefficient λ and the shear modulus is as follows:


$$\begin{array}{c} \lambda =\frac{2G_{\text{0}}\nu }{1-2\nu }=\frac{2\nu }{1-2\nu }\left[AF\left(Na_{\text{2}}SO_{\text{4}}\right)F\left(e\right)+B(\frac{\sigma^{\prime }}{P_{a}}{)}^{n}\right] \end{array}$$ (7)

Equation (7) quantifies the correlation between soil salinity and pile shaft shear stiffness coefficient λ, enabling settlement calculation of pile foundations under various salt contents.

## 4 Theoretical model of piles in saline soil foundations

### 4.1 Theoretical model of a single pile

**Fig 4:**
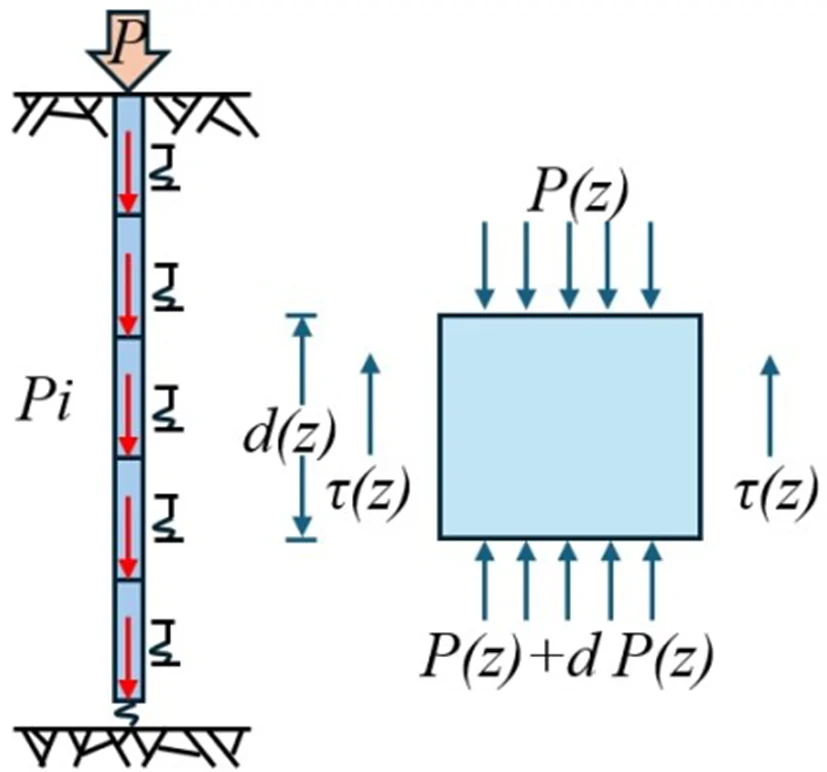
Force diagram of model pile element.

The load transfer method is a prevalent simplified approach for calculating the settlement of single piles. As proposed by [2], this method discretizes a pile into multiple segments along its longitudinal direction. A core assumption of this method is that the displacement at any position along the pile shaft is solely dependent on the local skin friction resistance at the corresponding location. Each discretized pile segment is modelled by an individual spring to characterize the pile–soil interaction, as illustrated in Fig 4.

For any pile element, the static equilibrium condition of the pile element can be expressed as:


$$\begin{array}{c} P\left(z\right)=\tau \left(z\right)dzU+P\left(z\right)+dP\left(z\right) \end{array}$$ (8)

Where: *U* is the perimeter of the pile cross-section; *τ(z)* is the lateral resistance at any point along the pile; *P(z)* is the axial force at any point along the pile. The compression of the pile element can be expressed as:


$$\begin{array}{c} ds=-\frac{P\left(z\right)+P\left(z\right)+dP\left(z\right)}{\text{2}}\frac{dz}{E_{p}A_{p}} \end{array}$$ (9)

Where: *E*_*P*_ is the elastic modulus of the pile; *A*_*P*_ is the cross-sectional area of the pile. Ignoring the second-order differential terms, the formula can be rewritten as follows:


$$\begin{array}{c} \frac{ds}{dz}=-\frac{P\left(z\right)}{E_{p}A_{p}} \end{array}$$ (10)

Taking the derivative of equation (10) and combining it with equation (9), we can obtain:


$$\begin{array}{c} \frac{d^{\text{2}}s}{dz^{\text{2}}}=\frac{U}{A_{P}E_{P}}\tau \left(z\right) \end{array}$$ (11)

**Fig 5:**
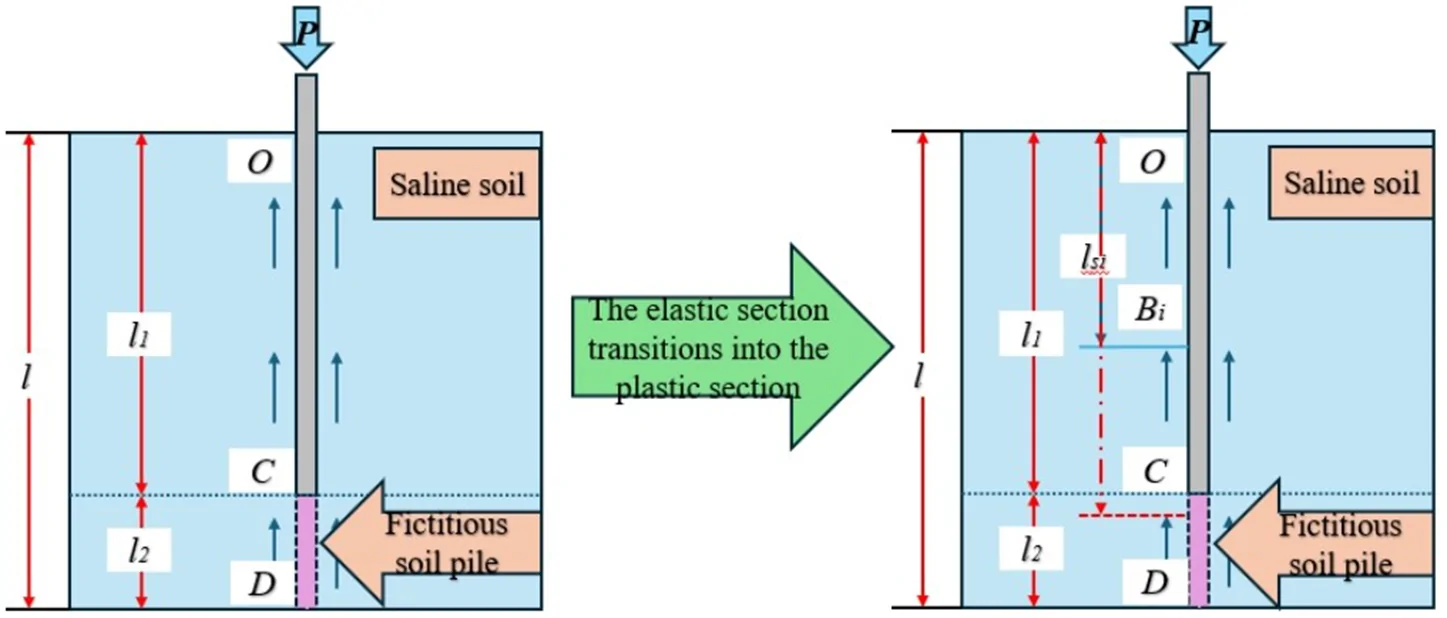
Diagrams of computational models at different stages.

Equation (11) is the governing differential equation for the load transfer method, whose analytical solution relies on the selected load transfer function. With rising pile head load, the surrounding soil undergoes an elastic-plastic transition. Under low loads, both the pile-side soil and fictitious soil pile behave elastically. As the load increases, plastic deformation initiates near the pile head and propagates downward along the pile shaft. Further loading induces full yielding of the surrounding soil and the formation of a plastic zone around the fictitious soil pile. This paper separately establishes three load transfer models corresponding to the three aforementioned loading stages. As shown in Fig 5.

#### (1) All the soil around the pile is in the elastic stage.

Using *P* and *S* to represent the pile head load and settlement, and *P*_*C*_ and *S*_*C*_ to represent the pile tip reaction force and displacement (i.e., the load and displacement at the top of the fictitious soil pile). When the pile head load is small, all the soil around the pile is in the elastic stage. The differential equation for the pile and the boundary conditions are as follows:


$$\begin{array}{c} \begin{array}{c} E_{P}A\frac{d^{\text{2}}s}{dz^{\text{2}}}-\lambda s=0\\ E_{P}A\frac{ds}{dz}|{\;}_{{z=l}_{\text{1}}}=-P_{C}\\ s|{\;}_{{z=l}_{\text{1}}}=s_{C} \end{array}\} \end{array}$$ (12)

The differential equation and boundary conditions for the fictitious soil pile are as follows:


$$\begin{array}{c} \begin{array}{c} E_{s}A\frac{d^{\text{2}}s}{dz^{\text{2}}}-\lambda s=0\\ E_{s}A\frac{ds}{dz}|{\;}_{z=l_{\text{1}}}=-P_{C}\\ s|{\;}_{z=l_{\text{1}}}=s_{C}\\ s|{\;}_{z=l_{\text{1}}+l_{\text{2}}}=0 \end{array}\} \end{array}$$ (13)

In equation (13), *E*_*p*_, *E*_*s*_, *A*, and *λ* represent the elastic modulus of the pile, the elastic modulus of the fictitious soil pile, the cross-sectional area of the pile, and the shear stiffness coefficient of the soil around the pile, respectively. In the equations: $b_{\text{1}}=\sqrt{\lambda /E_{p}A},b_{\text{2}}=\sqrt{\lambda /E_{s}A}$, Solving equations (12) and (13):


$$\begin{array}{c} \begin{array}{c} \begin{array}{cc} s\left(z\right)=\frac{b_{\text{1}}s_{{\;}_{C}}-\frac{p_{C}}{E_{p}A}}{2b_{\text{1}}e^{b_{\text{1}}l_{\text{1}}}}e^{b_{\text{1}}z}+\frac{b_{\text{1}}s_{C}+\frac{p_{C}}{E_{p}A}}{2b_{\text{1}}e^{-b_{\text{1}}l_{\text{1}}}}e^{-b_{\text{1}}z} & 0\leq z \end{array}<l_{\text{1}}\\ \begin{array}{cc} s\left(z\right)=\frac{b_{\text{2}}s_{C}-\frac{p_{C}}{E_{s}A}}{2b_{\text{2}}e^{b_{\text{2}}l_{\text{1}}}}e^{b_{\text{2}}z}+\frac{b_{\text{2}}s_{C}+\frac{p_{C}}{E_{s}A}}{2b_{\text{2}}e^{-b_{\text{2}}l_{\text{1}}}}e^{-b_{\text{2}}z} & l_{\text{1}}\leq z \end{array}\leq l_{\text{2}} \end{array}\} \end{array}$$ (14)

Based on the displacement at the bottom of the fictitious soil pile being zero, it is known that: $s\left(z\right)|{\;}_{z=l_{\text{1}}+l_{\text{2}}}=0$.


$$\begin{array}{c} P_{C}=E_{s}A\xi b_{\text{2}}s_{C} \end{array}$$ (15)

Where: $\xi =\frac{e^{2b_{\text{2}}l_{\text{2}}}+1}{e^{2b_{\text{2}}l_{\text{2}}}-1}$, Let$K_{C}=E_{s}A\xi b_{\text{2}}$, Then the relationship between the axial force at the pile tip and the load at the pile tip is:


$$\begin{array}{c} P_{C}=K_{C}s_{C} \end{array}$$ (16)

The load-settlement relationship at the pile head is:


$$\begin{array}{c} \begin{array}{c} P_{\text{0}}=-E_{p}A\frac{ds}{dz}|{\;}_{z=0}=-E_{p}A\left(\frac{b_{\text{1}}s_{C}-\frac{p_{C}}{E_{p}A}}{2e^{b_{\text{1}}l_{\text{1}}}}-\frac{b_{\text{1}}s_{C}+\frac{p_{C}}{E_{p}A}}{2e^{-b_{\text{1}}l_{\text{1}}}}\right)\\ s_{\text{0}}=s|{\;}_{z=0}=\frac{b_{\text{1}}s_{C}-\frac{p_{C}}{E_{p}A}}{2b_{\text{1}}e^{b_{\text{1}}l_{\text{1}}}}+\frac{b_{\text{1}}s_{C}+\frac{p_{C}}{E_{p}A}}{2b_{\text{1}}e^{-b_{\text{1}}l_{\text{1}}}} \end{array}\} \end{array}$$ (17)

Based on equation (17), the pile head stiffness *K*_*0*_ can be obtained as:


$$\begin{array}{c} K_{\text{0}}=b_{\text{1}}E_{p}A\frac{K_{C}+b_{\text{1}}E_{p}Atanh\left(b_{\text{1}}l_{\text{1}}\right)}{b_{\text{1}}E_{p}A+K_{C}tanh\left(b_{\text{1}}l_{\text{1}}\right)} \end{array}$$ (18)

According to Equation (18), for a given pile-soil system, when the soil around the pile is entirely in the elastic stage, the *P*-*S* curve is a straight line segment with a slope of *K*_*0*_.

#### (2) The soil around the pile partially enters the plastic state.

When the pile head settlement exceeds the ultimate displacement *S*_*b*_, if the pile head load continues to increase, the soil around the pile will yield layer by layer from the bottom up, forming a plastic zone from shallow to deep. At this time, it is necessary to re-establish the differential control equation for the plastic segment and provide the corresponding boundary conditions.


$$\begin{array}{c} \begin{array}{c} E_{P}A\frac{d^{\text{2}}s}{dz^{\text{2}}}-\lambda s_{b}=0\\ E_{P}A\frac{ds}{dz}|{\;}_{z=l_{s}}=-P_{B}\\ s|{\;}_{z=l_{s}}=s_{B} \end{array}\} \end{array}$$ (19)

The solution of Equation (19) yields:


$$\begin{array}{c} \begin{array}{cc} s\left(z\right)=\frac{\text{1}}{\text{2}}\frac{\lambda s_{b}}{E_{p}A}z^{\text{2}}-\frac{\lambda s_{b}l_{s}+P_{B}}{E_{p}A}z+s_{B}+\frac{P_{B}l_{s}}{E_{p}A}\text{+}\frac{\text{1}}{\text{2}}\frac{\lambda s_{b}}{E_{p}A}l_{s}^{\text{2}}, & 0\leq z\leq \end{array}l_{\text{1}} \end{array}$$ (20)

From the above equation, the pile head load and pile head settlement can be obtained as:


$$\begin{array}{c} \begin{array}{c} P_{\text{0}}=-E_{P}A\frac{ds}{dz}|{\;}_{z=0}=P_{B}+\lambda s_{b}l_{s}\\ s_{\text{0}}=s|{\;}_{z=0}=s_{B}+\frac{P_{B}l_{s}}{E_{p}A}+\frac{\text{1}}{\text{2}}\frac{\lambda s_{b}l_{s}^{\text{2}}}{E_{p}A} \end{array}\} \end{array}$$ (21)

In Equation (21):


$$\begin{array}{c} P_{B}=K_{B}s_{B} \end{array}$$ (22)

$$\begin{array}{c} K_{B}=b_{\text{1}}E_{p}A\frac{b_{\text{2}}E_{s}A\xi +b_{\text{1}}E_{p}Atanh\left[b_{\text{1}}(l_{\text{1}}-l_{a})\right]}{b_{\text{1}}E_{p}A+b_{\text{2}}E_{s}A\xi tanh\left[b_{\text{1}}(l_{\text{1}}-l_{a})\right]} \end{array}$$ (23)

The pile head stiffness is:


$$\begin{array}{c} K_{\text{1}}=\frac{P_{\text{0}}}{s_{\text{0}}}=-\frac{\frac{P_{B}+\lambda s_{b}l_{s}}{E_{p}A}}{s_{B}+\frac{P_{B}l_{s}}{E_{p}A}+\frac{\text{1}}{\text{2}}\frac{\lambda s_{b}l_{s}^{\text{2}}}{E_{p}A}} \end{array}$$ (24)

#### (3) The soil around the pile partially enters the slip stage, that is, the virtual soil pile partially enters the plastic stage.

As the load at the top of the pile continues to increase, the soil around the pile reaches a plastic state, and the virtual soil pile begins to enter the plastic stage.

For the *OC* section of the pile, the differential equation and boundary conditions are:


$$\begin{array}{c} \begin{array}{c} E_{P}A\frac{d^{\text{2}}s}{dz^{\text{2}}}-\lambda s_{b}=0\\ E_{P}A\frac{ds}{dz}|{\;}_{{z=l}_{\text{1}}}=-P_{C}\\ s|{\;}_{z=l_{\text{1}}}=s_{C} \end{array}\} \end{array}$$ (25)

For the plastic segment *CB* of the virtual soil pile, the differential equation and boundary conditions are:


$$\begin{array}{c} \begin{array}{c} E_{s}A\frac{d^{\text{2}}s}{dz^{\text{2}}}-\lambda s_{b}=0\\ E_{s}A\frac{ds}{dz}|{\;}_{z=l_{s}}=-P_{B}\\ s|{\;}_{{z=l}_{s}}=s_{B} \end{array}\} \end{array}$$ (26)

For the elastic segment *BD* of the virtual soil pile, the differential equation and boundary conditions are:


$$\begin{array}{c} \begin{array}{c} E_{s}A\frac{d^{\text{2}}s}{dz^{\text{2}}}-\lambda s=0\\ E_{s}A\frac{ds}{dz}|{\;}_{z=l_{s}}=-P_{B}\\ \begin{array}{cc} s|{\;}_{{z=l}_{s}}=s_{B}, & s|{\;}_{z=l_{\text{1}}+l_{\text{2}}}=0 \end{array} \end{array}\;\;\;\;\;\;\} \end{array}$$ (27)

The solution to the above equation is:


$$\begin{array}{c} P_{B}=b_{\text{2}}E_{s}A\xi_{\text{3}}s_{B} \end{array}$$ (28)

Where: $\xi_{\text{3}}=\frac{e^{2b_{\text{2}}(l-l_{s})}+1}{e^{2b_{\text{2}}(l-l_{s})}-1}$. Using the principle of equal displacement and force at the pile-soil cross-section, the axial force and displacement at the pile base section *C* are obtained as follows:


$$\begin{array}{c} \begin{array}{c} P_{C}=P_{B}+\lambda s_{b}(l_{s}-l_{\text{1}})\\ s_{C}=s_{B}+\frac{P_{B}l_{s}}{E_{s}A}+\frac{\text{1}}{\text{2}}\frac{\lambda s_{b}}{E_{s}A}(l_{s}^{\text{2}}+l_{\text{1}}^{\text{2}})-\frac{P_{B}+\lambda s_{b}l_{s}}{E_{s}A}l_{\text{1}} \end{array}\;\;\;\} \end{array}$$ (29)

The pile head load and pile head settlement can further be obtained as:


$$\begin{array}{c} \begin{array}{c} P_{\text{0}}=P_{C}+\lambda s_{b}l_{\text{1}}\\ s_{\text{0}}=s_{C}+\frac{P_{C}l_{\text{1}}}{E_{P}A}+\frac{\text{1}}{\text{2}}\frac{\lambda s_{b}}{E_{P}A}l_{\text{1}}^{\text{2}} \end{array}\;\;\;\} \end{array}$$ (30)

Then the pile head stiffness is:


$$\begin{array}{c} K_{\text{2}}=\frac{P_{\text{0}}}{s_{\text{0}}}=\frac{P_{C}+\lambda s_{b}l_{\text{1}}}{s_{C}+\frac{P_{C}l_{\text{1}}}{E_{P}A}+\frac{\text{1}}{\text{2}}\frac{\lambda s_{b}}{E_{P}A}l_{\text{1}}^{\text{2}}} \end{array}$$ (31)

### 4.2 Vertical stiffness of pile groups

According to [37], the theoretical calculation methods for single piles and pile groups were obtained. The interaction coefficient of pile groups is defined as $\alpha_{ij}$, and its expression is as follows:


$$\begin{array}{c} \alpha_{ij}=\frac{\delta_{ij}}{\delta_{ii}} \end{array}$$ (32)

Where: *α*_*ij*_ is the interaction coefficient of pile groups, *δ*_*ij*_ is the settlement of pile *j* when a unit load is applied to pile *i*, and *δ*_*ii*_ is the settlement of pile *i* when a unit load is applied to pile *i*.

Under vertical loading, the stiffness *K*_*Q*_ is the ratio of the total force *F*_*Q*_ at the pile group head to the displacement *S*_*Q*_ at the pile group head, and its expression is as follows:


$$\begin{array}{c} K_{Q}=\frac{F_{Q}}{S_{Q}} \end{array}$$ (33)

The total displacement of the pile group is equal to the displacement of any single pile within it, while the total load on the pile group is the sum of the loads at the tops of each individual pile. Therefore, we can obtain: Through Equations (32) and (33), the stiffness of the pile group can be obtained as:


$$\begin{array}{c} K_{11}=\frac{F_{Q}}{S_{11}\sum_{m=1}^{N}\alpha_{m1}} \end{array}$$ (34)

Substituting *F*_*Q*_ = *n F* into Equation (35), the stiffness of the pile group can be obtained as:


$$\begin{array}{c} K_{Q}=\frac{nF}{W_{Q}}=\frac{nK_{\text{0}}}{S_{11}\sum_{m=1}^{N}\alpha_{m1}} \end{array}$$ (35)

The simplified calculation methods for the vertical stiffness of single piles and pile groups can be obtained from Equations (32) and (35). The vertical stiffness of a pile group is not equal to the product of the vertical stiffness of a single pile and the number of piles. To more specifically investigate the efficiency of pile groups, the pile group efficiency coefficient *η* is defined as the ratio of the pile head stiffness of the pile group to that of a single pile, and its expression is:


$$\begin{array}{c} \eta =\frac{K_{Q}}{nK_{\text{0}}} \end{array}$$ (36)

## 5 Result analysis

### 5.1 Comparison of experimental results with theoretical results

**Fig 6:**
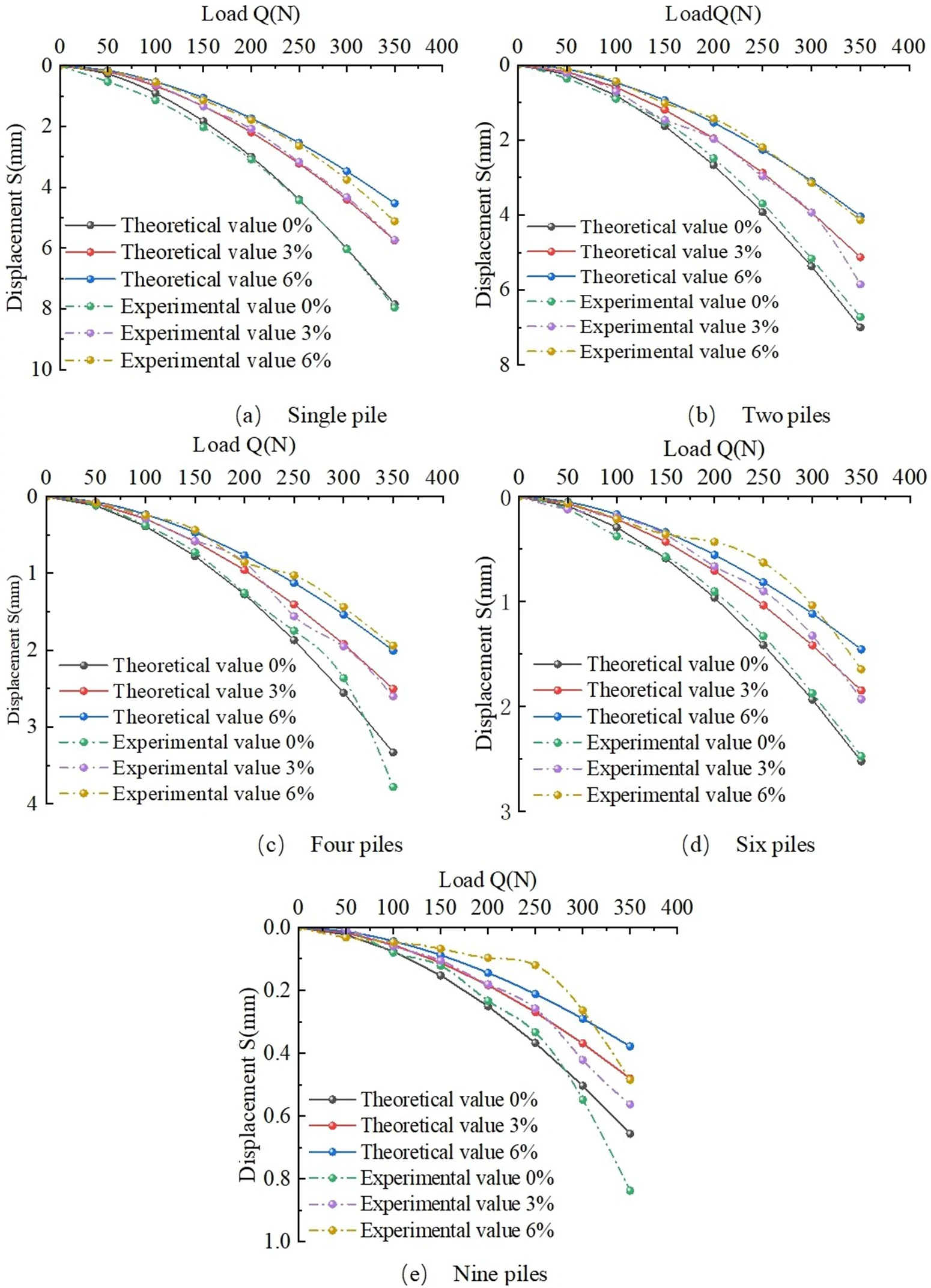
Comparison of Q-S Curves between experimental and theoretical results of pile group tests under different salt content conditions. (a) Single pile; (b) Two piles; (c) Four piles; (d) Six piles; (e) Nine piles.

To verify the correctness of the results in this paper, based on the *Q*-*S* curve shown in Fig 6, the comparison and analysis of the experimental values and theoretical values indicate that the theoretical calculation results of single pile, double pile, four pile, six pile, and nine pile are basically in agreement with the experimental data.

As depicted in Fig 6, the displacement of all pile foundation specimens rises monotonically with the applied load. Noticeable discrepancies exist in both the magnitude and profile of the load-displacement curves corresponding to soils with varying salt contents (0%, 3%, and 6%). At identical load magnitudes, pile displacement gradually declines as the salt concentration of the saline soil increases. This observation reveals that salt incorporation exerts a reinforcing effect on the mechanical behaviour of the host soil. The above trend aligns with the findings reported by previous researchers [38,39], who stated that soil strength increases monotonically with rising salt content under low-salinity environments.

In addition, as the number of piles increases, the settlement of the pile group under the same load is significantly less than that of a single pile and does not show a linear relationship. This reflects that the interaction effect between piles becomes stronger with the increase in the number of piles, and the pile group effect becomes more pronounced.

### 5.2 Pile foundation stiffness variation analysis

It can be known through analysis that under the test conditions with salt contents of 0%, 3%, and 6% respectively, the pile head stiffness of single pile, double pile, four piles, six piles, and nine piles all show a significant linear growth trend, and the pile head stiffness increases gradually with the increase of pile foundation quantity. Among them, in the saline soil with different salt contents, the pile head stiffness of single pile is always at the lowest level, while that of nine piles is the highest.

**Fig 7:**
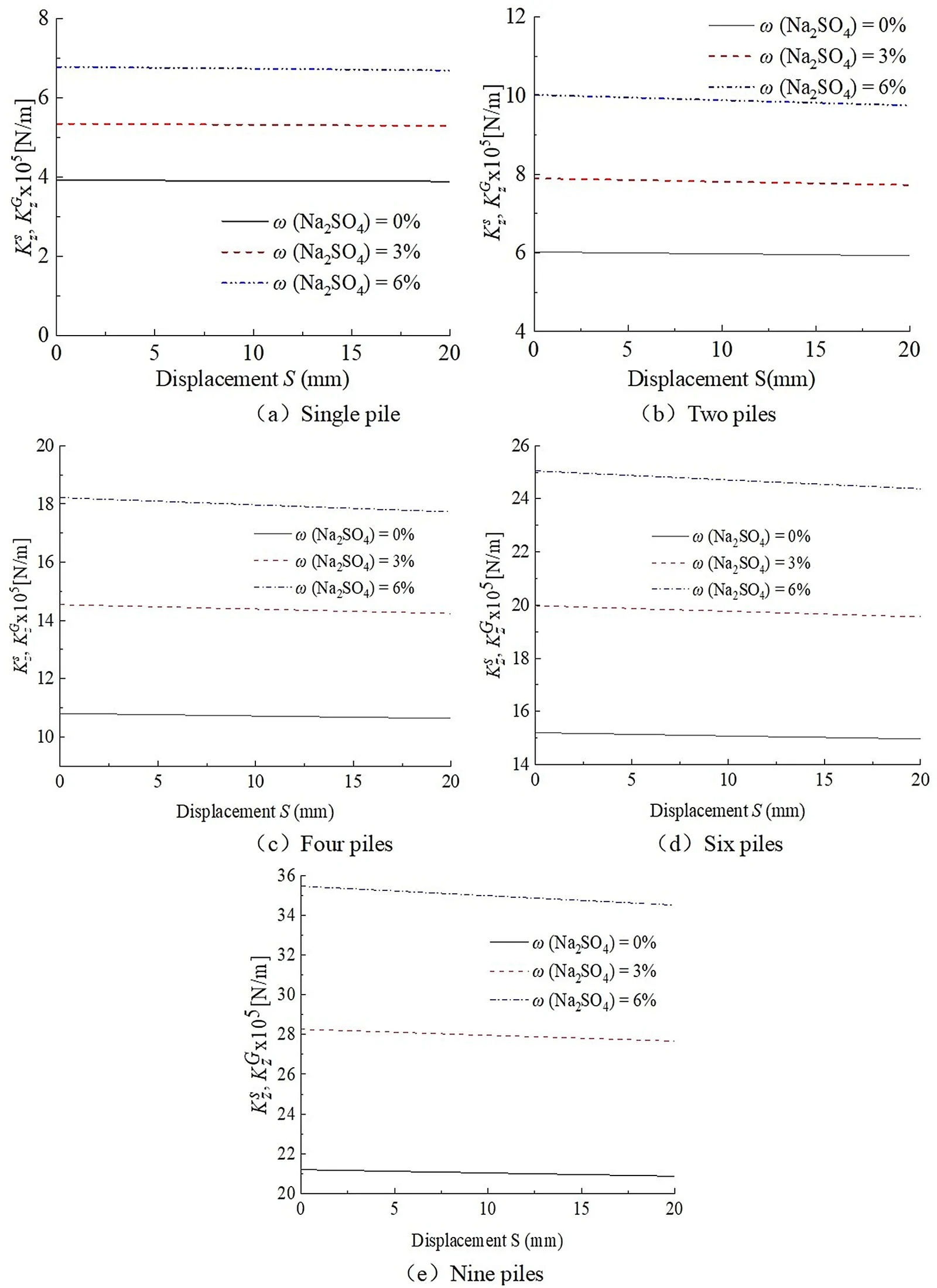
Experimental results of pile head stiffness under different salinity conditions. (a) Single pile; (b) Two piles; (c) Four piles; (d) Six piles; (e)Nine piles.

In addition, as shown in Fig 7, the ratio of the pile head stiffness of the double pile to that of the single pile varies between 1.47 and 1.5. The ratio of the pile head stiffness of the four piles to that of the single pile varies between 2.7 and 2.8. The ratio of the pile head stiffness of the six piles to that of the single pile varies between 3.6 and 3.9. The ratio of the pile head stiffness of the nine piles to that of the single pile varies between 5.2 and 5.4. This demonstrates that the interaction between piles has a significant impact on the overall stiffness of the pile foundation; the pile head stiffness of the pile group is not a multiple of the stiffness of the single pile.

### 5.3 Group pile effect analysis

In order to consider the bearing influence of the group pile effect on different pile foundations, this experiment was carried out and the experimental results were analyzed. It was found that the theoretical calculation results basically coincide with the experimental data, indicating that the experiment has good reliability.

**Fig 8:**
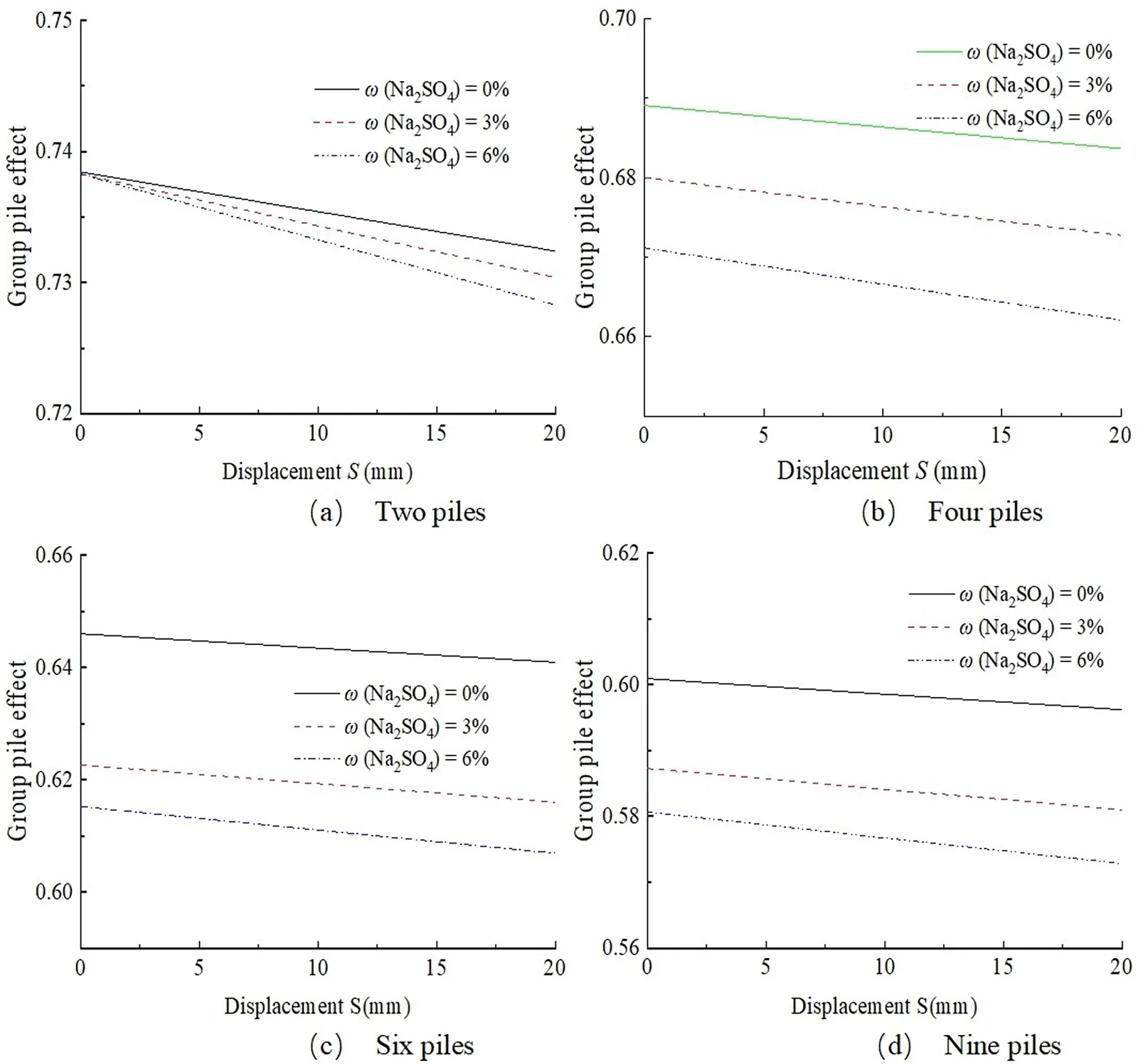
Experimental results of group piling effect *η* under different salinity conditions. (a) Two piles; (b) Four piles; (c) Six piles; (d) Nine piles.

As shown in Fig 8, under the conditions of salt content of 0%, 3%, and 6%, the group pile effect coefficient of the double pile is the largest, with a numerical range of 0.738 to 0.727; the group pile effect coefficient of the nine piles is the smallest, with a numerical range of 0.6 to 0.57. In addition, with the increase of salt content, the group pile effect coefficient of different pile types shows a significant downward trend. This is because the salt content has a significant impact on the group pile effect, and the higher the salt content, the stronger the group pile effect. At the same time, as the number of piles increases, the interaction mechanism between piles becomes more complex, and the degree of mutual influence is significantly enhanced, which in turn leads to a decrease in the group pile effect coefficient.

In addition, it can be known through the analysis in combination with Fig 8 that the group pile effect curve exhibits linear characteristics and undergoes dynamic changes with the development of displacement. The reason for the formation of this pattern is that under large displacement conditions, higher salt content will further weaken the group pile effect, while the interaction effect between pile foundations becomes more prominent.

### 5.4 Analysis of the influence of axial force on the pile shaft

On-site testing is unable to directly measure the axial force of the pile body. Therefore, it is necessary to rely on the pile body’s deformation changes to calculate the axial force distribution of the pile body under various loads. Based on the theory of axial deformation and stress of the bar, the following formula is used to calculate the axial force at any section of the pile body under vertical load:


$$\begin{array}{c} N=\varepsilon_{avg}E\cdot A \end{array}$$ (37)

In the formula: *ε*_*avg*_*=*(ε_*Left*_+ε_*Right*_)/2, ε_*Left*_ represents the strain value on the left side of the pile body, ε_*Right*_ represents the strain value on the right side of the pile body, and E represents the elastic modulus of the pile body. A represents the cross-sectional area of the pile body.

**Fig 9:**
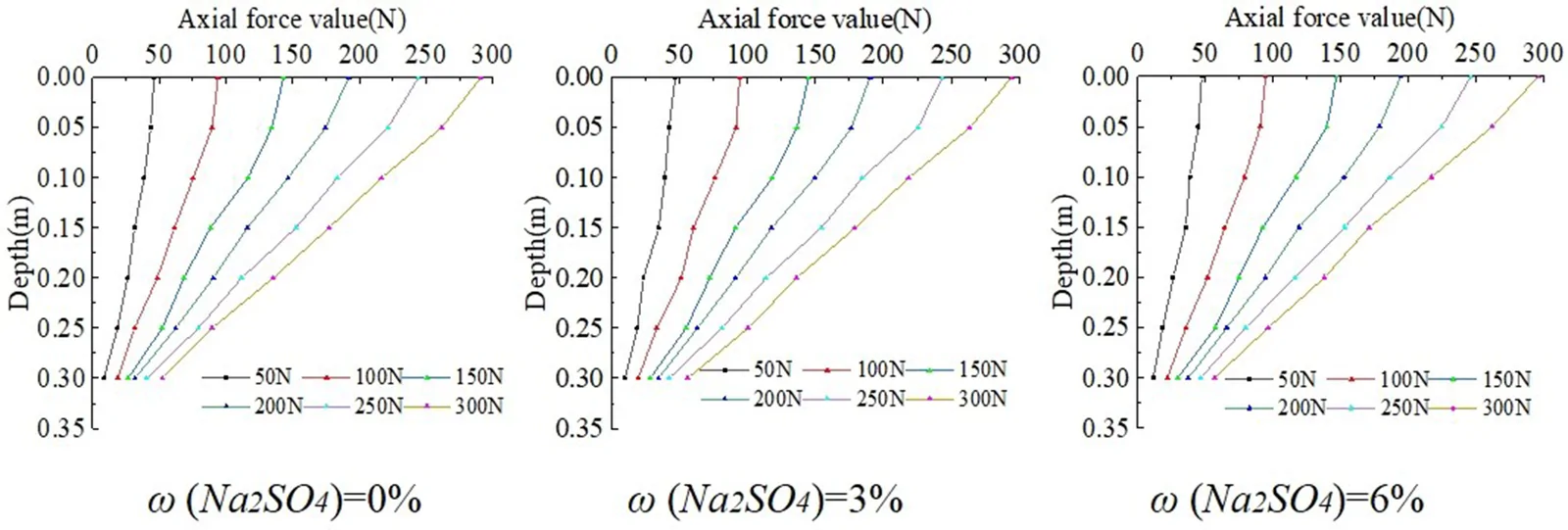
Shows the variation law of the axial force values of the pile body of Angle piles with different salt contents. (a) Corner pile.

**Fig 10:**
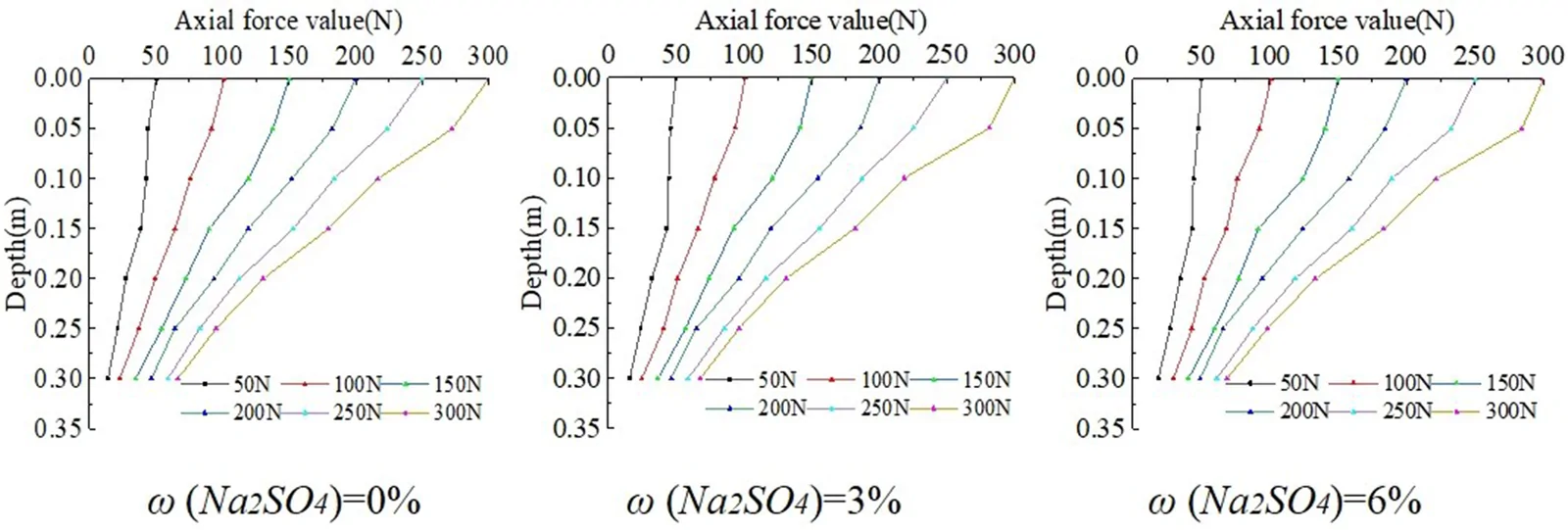
Shows the variation law of the axial force values of the pile body of edge piles with different salt contents. (b) Side pile.

**Fig 11:**
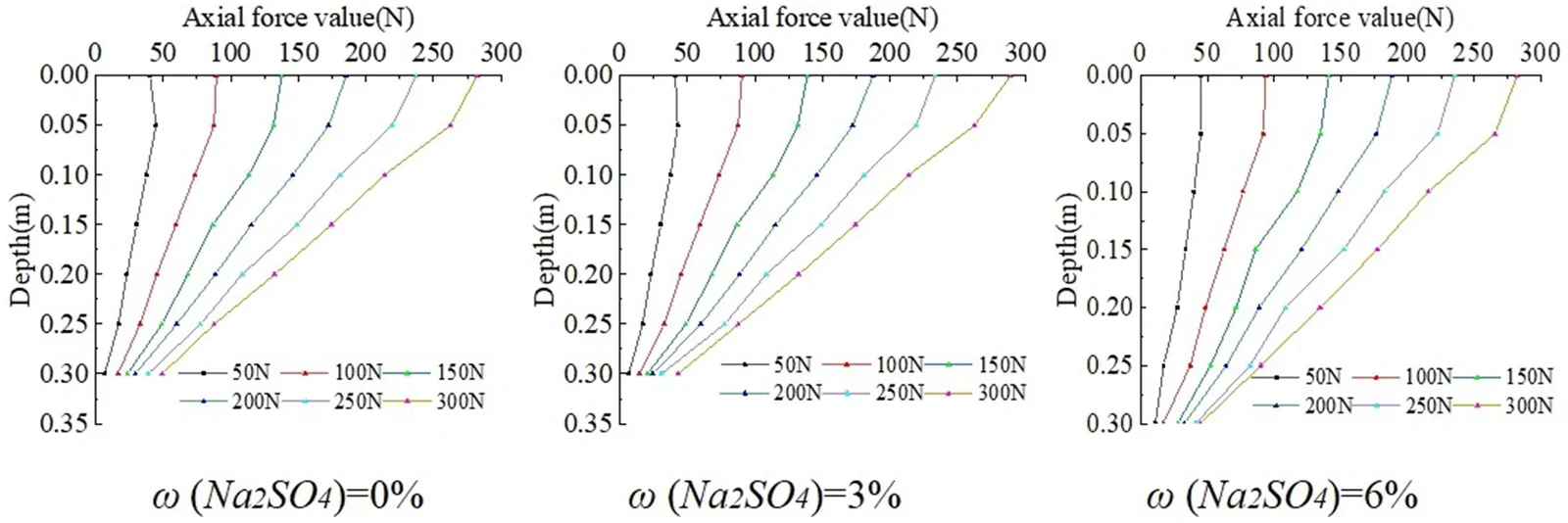
Shows the variation law of the axial force values of the pile shaft of central piles with different salt contents. (c) Central pile.

Figs 9–11 presents the variation law of the axial force values of the pile shafts of the corner piles, edge piles and center piles of nine piles under different salt contents. It can be found from the figure that as the vertical load gradually increases, the axial forces of the pile shafts of the corner piles, edge piles and center piles all increase.

Furthermore, according to Figs 9–11, it is found that the axial force value of the pile body decreases as the depth increases. With the increase of salt content, the axial force values of corner piles, center piles and edge piles increase accordingly. This is because the salt re-cements the soil particles, enhancing the stiffness and strength of the soil, and the lateral frictional resistance increases accordingly, resulting in an increase in the cumulative axial force value of the pile body.

In addition, under the same level of load, compared with corner piles and edge piles, the axial force of the central pile body is the smallest, followed by the edge pile, and the corner pile is the largest. This is because the central pile experiences the least impedance due to the group pile effect, bears less load, and the pile-pile and pile-soil interactions are significant. The corner piles are the farthest from the center, the retaining effect of the group piles is weak, and they share more loads.

## 6 Conclusions

In this paper, the bearing performance tests of pile foundations under different salt contents were conducted. When parameters such as the shear stiffness coefficient of saline soil changed, the test results of this paper were combined with theory to analyze the *Q*-*S* curves of each pile foundation, the stiffness of the pile head, the group pile effect and the variation laws of the axial force of the pile body, and the following conclusions were drawn:

(1) This paper combines the load transfer method with the pseudo-soil pile theory, establishing a force model for a single pile in saline soil, and proposing an explicit relationship formula between “salt content, shear stiffness and the vertical bearing capacity of multiple piles.” The theoretical solution shows a very small error compared with the model test results. Moreover, the settlement algorithm of the pseudo-soil pile has been extended to be applicable to pile groups, thereby deriving the settlement calculation formula for multiple piles. This method provides a valuable research path for predicting the bearing capacity of pile foundations in saline soil based on measured data.
(2) The variation in shear stiffness with respect to salt content has a direct impact on soil strength, which subsequently influences the vertical bearing capacity of pile foundations. In environments characterized by increased salt content, both the stiffness at the pile head and the *Q*-*S* curves exhibit significant improvements as the number of piles increases, leading to a notable enhancement in the bearing efficiency of the pile group. Furthermore, it is observed that the pile head stiffness for different types of pile foundations does not simply scale as a multiple of that for a single pile when considering varying numbers of piles.
(3) The analysis of the group pile effect coefficient indicates that, under conditions of substantial displacement, an increased salt content further diminishes the group pile effect. Furthermore, as the number of piles increases, the mutual interactions among them become more pronounced.
(4) Analysis of axial force distributions for corner, edge, and central piles within the 9-pile group reveals that axial forces of all piles rise with increasing load and salt concentration, yet decline with depth. At equal applied loads, axial forces follow the order: corner pile > edge pile > central pile. Such differences stem from pile group shielding effects and pile–soil interaction mechanisms.
(5) This test is a short-term constant temperature and humidity static load test. It does not take into account the effects of dry-wet cycles, long-term ion erosion, repeated dissolution and crystallization of salts, etc., which are all time-averaging effects. It only focuses on the instantaneous shear mechanical behavior of the soil skeleton and does not include the long-term deterioration and evolution process of the pile material. The research conclusion is only applicable to the static and humidity-stable short-term loading scenario and does not represent the performance of the entire life cycle engineering.

## References

[1] Zhou Z, Jin Q, Gong J, Li Z. Enhancement mechanisms of corrosion resistance in steel slag sand concrete under saline soil environments: pore structure optimization and alkalinity regulation. Construct Building Mat. 2026;521:146137. doi: 10.1016/j.conbuildmat.2026.146137

[2] Seed HB, Reese LC. The Action of Soft Clay along Friction Piles. T Am Soc Civ Eng. 1957;122(1):731–54. doi: 10.1061/taceat.0007501

[3] Kezdi A. The bearing capacity of pile and pile groups. In: Proceedings of the 4th International Conference on Soil Mechanics and Foundations Engineering, London. 1957. 46–51. https://www.issmge.org/uploads/publications/1/41/1957_02_0010

[4] Coyle HM, Reese LC. Load transfer for axially loaded piles in clay. J Soil Mech and Found Div. 1966;92(2):1–26. doi: 10.1061/jsfeaq.0000850

[5] Vijayvergiya VN. Load-movement characteristics of piles. In: Proc. of 4th Symposium of Waterway, Port, Coastal and Ocean Division, ASCE, Long Beach, Calif., 1977.

[6] Fang W, Zhao M, Su J. Analysis of the vertical ultimate bearing capacity of piles controlled by settlement. Central South Highway Eng. 1999;25–7.

[7] Pan S. Analysis of load transfer of piles by layered integration method. J Build Struct. 1991;(05):68–79.

[8] Zhao M hua, Zou D, Zou X. Research on settlement calculation method of high pile cap foundation based on load transfer method. Chinese J Rock Mech Eng. 2005;2005(13):2310–4.

[9] Zhao M, Zou D, Zou X. Load transfer method for settlement calculation of group piles. Eng Mech. 2006;(07):119–23.

[10] Wang T, Zhang Y, Zheng J. Long-term response of energy piles based on the hyperbolic tangent model. J Central South University (Science and Technology). 2024;55(12):4593–603. doi: 10.11817/j.issn.1672-7207.2024.12.018

[11] Ma W, Wang X u, Wang B, et al. Study on the transformation of load-settlement curve based on load transfer at pile-soil interface. Chinese J Geotech Eng. 2021;43(S1):41–6. doi: 10.11779/CJGE2021S1008

[12] Gao M, Gao G, Yang C, Feng S, Ji Y. Analytical solution of shear displacement for settlement calculation of group piles in layered foundation. Rock and Soil Mechanics. 2010;31(4):1072–7.

[13] Cooke RW, Price G, Tarr K. Jacked piles in London clay: a study of load transfer and settlement under working conditions. Geotechnique. 1979;29(2):113–47. doi: 10.1680/geot.1979.29.2.113

[14] Ye S, Xin L. Study on settlement and bearing capacity of single pile based on shear characteristics of pile-soil interface. Rock and Soil Mech. 2024;45(05):1457–71.

[15] Randolph MF, Wroth CP. Analysis of deformation of vertically loaded piles. J Geotech Engrg Div. 1978;104(12):1465–88. doi: 10.1061/ajgeb6.0000729

[16] Poulos HG. Analysis of settlement of pile groups. Geotechnique. 1968;18(3):449–71. doi: 10.1680/geot.1968.18.4.449

[17] Chen Z, Chen F, Weng Y. Calculation method of vertical bearing capacity of large diameter steel pipe piles considering soil plug effect. Rock and Soil Mech. 2025;46(07):2224–36. doi: 10.16285/j.rsm.2024.1306

[18] Wang K, Lv S, Wu W. Settlement calculation method of single piles in layered foundation based on the virtual soil pile model. Eng Mech. 2013;30(07):75–83. doi: 10.6052/j.issn.1000-4750.2012.03.0171

[19] Dong C. Research on settlement theory and engineering application of pile foundations based on the virtual soil pile method. Central South University; 2023.

[20] Li J, Zhao G, Shi M. Degradation and life prediction model for piles due to crystallisation attack in sulfate saline area. Adv Cement Res. 2020;32(4):181–95. doi: 10.1680/jadcr.18.00147

[21] Shao W, Shi D. Numerical simulation of degradation behavior of concrete piles in sulfate saline soils. KSCE J Civil Eng. 2022;26(1):183–92. doi: 10.1007/s12205-021-2225-9

[22] Sun J, Yu H, Wu C, et al. Long-term damage and deterioration laws of corroded concrete in Chaerhan Salt Lake. Construct Materials J. 2025;28(10):980–5.

[23] Zuo S, Zhang X, Lu X, et al. Reliability analysis of vertical bearing capacity of bridge pile foundations in permafrost areas considering parameter variability. Eng Sci Tech. 2025;1–11. doi: 10.12454/j.jsuese.202400823

[24] Cui M. Research on the influence of saline soil foundation improvement on the load-bearing characteristics of bridge pile foundations in permafrost regions. Lanzhou Jiaotong University; 2024.

[25] Deng Y, Chen C, Yang Q, et al. Model test study on the influence of pile spacing on the bearing characteristics of group piles. Eng Sci Tech. 2026;1–13. doi: 10.12454/j.jsuese.202500595

[26] Cao X, Zhou F, Dai G, et al. Study on the vertical vibration characteristics of frictional single piles and group piles in multi-layer soils. Chinese J Geotech Eng. 2025;47(9):1856–64. doi: 10.11779/CJGE20240442

[27] Lyu W, Wang Z, Zhao X. Numerical simulation of heat transfer in saline soil energy pile groups. Energies. 2026;19(11):2725. doi: 10.3390/en19112725

[28] Yang X, Wang D, Zhang S, et al. Deformation characteristics of long and short pile composite foundation for high-speed railway in Yanyu district. J Transport Eng. 2024;24(3):181–92.

[29] Rao SN, Singh H, Kumar S. Boundary effects in pile load tests on model piles in clay. Canadian Geotechnical J. 2000;37(6):1298–305.

[30] Guo C, Gong W, Lu B, et al. Experimental study on the relative stiffness of pile-raft foundation. Chinese J Geotech Eng. 2008;30(12):1840–6.

[31] Bing H, Wu J u n j i e, Deng J i n. Changes in the physical and mechanical properties of loess-like saline soil before and after desalination. Journal of Glaciology and Geocryology. 2011;33(4):796–800. doi: 10.7522/j.issn.1000-0240.2011.0106

[32] Code for design of foundation of buildings (GB50007-2011). Beijing: China Architecture & Building Press; 2012.

[33] Wang X, Xia T, Zhang L, et al. Experimental study on dynamic properties at small strain and microstructure of saline soil. J Railway Eng Society. 2023;40(05):1–7.

[34] Ji Y, Bai B, Li X, Cui H. Influence of cooling-induced salt crystallization on the shear strength of sulfate saline soils. Cold Regions Sci Tech. 2025;240:104644. doi: 10.1016/j.coldregions.2025.104644

[35] Hardin BO, Black WL. Sand stiffness under various triaxial stresses. J Soil Mech and Found Div. 1966;92(2):27–42. doi: 10.1061/jsfeaq.0000865

[36] Ding S, Bi Q. Experimental study on the shear modulus of rockfill material. Yellow River. 2012;34(08):141–3. doi: 10.3969/j.issn.1000-1379.2012.08.049

[37] Cao X, Zhou F, Dai G, et al. Study on vertical vibration characteristics of single friction piles and group piles in multi-layered soil. Chinese J Geotech Eng. 2025;47(09):1856–64. doi: 10.11779/CJGE20240442

[38] Ma H, Chang L. Influence of water and salt content on the strength and permeability characteristics of loess-like saline soil in cold and arid regions. Sci Tech Eng. 2023;23(28):12193–203.

[39] Bing H, He P. Experimental study on the redistribution law of water and salt in saline soil under different freezing methods. Rock and Soil Mech. 2011;32(08):2307–12.

